# The long-term costs for treating multiple sclerosis in a 16-year retrospective cohort study in Brazil

**DOI:** 10.1371/journal.pone.0199446

**Published:** 2018-06-21

**Authors:** Isabela Maia Diniz, Augusto Afonso Guerra, Livia Lovato Pires de Lemos, Kathiaja M. Souza, Brian Godman, Marion Bennie, Björn Wettermark, Francisco de Assis Acurcio, Juliana Alvares, Eli Iola Gurgel Andrade, Mariangela Leal Cherchiglia, Vânia Eloisa de Araújo

**Affiliations:** 1 SUS Collaborating Centre for Technology Assessment & Excellence in Health, Faculdade de Farmácia, Universidade Federal de Minas Gerais, Belo Horizonte, Minas Gerais, Brazil; 2 Programa de Pós-Graduação em Medicamentos e Assistência Farmacêutica, Faculdade de Farmácia, Universidade Federal de Minas Gerais, Belo Horizonte, Minas Gerais, Brazil; 3 Programa de Pós-Graduação em Saúde Pública, Faculdade de Medicina, Universidade Federal de Minas Gerais, Belo Horizonte, Minas Gerais, Brazil; 4 Departamento de Gestão e Incorporação de Tecnologias em Saúde, Secretaria de Ciência, Tecnologia e Insumos Estratégicos, Ministério da Saúde, Esplanada dos Ministérios Bloco G, Brasília, Distrito Federal, Brazil; 5 Programa de Pós-Graduação em Saúde Baseada em Evidências, Universidade Federal de São Paulo, São Paulo, São Paulo, Brazil; 6 Strathclyde Institute of Pharmacy & Biomedical Sciences, Pharmacoepidemiology, Strathclyde University, Glasgow, United Kingdom; 7 Division of Clinical Pharmacology. Karolinska Institute, Stockholm, Sweden; 8 Division of Clinical Pharmacology, Karolinska Institutet, Karolinska University Hospital, Stockholm, Sweden; Universita degli Studi di Napoli Federico II, ITALY

## Abstract

**Background:**

Multiple Sclerosis (MS) is a disease that appreciably impacts on the quality of life of patients and is associated with high expenditure. MS is a chronic multifactorial disease, characterized by inflammation, demyelination and axonal loss. The Brazilian public health system provides pharmacological treatment as well as hospital and outpatient care for patients with relapsing-remitting and secondary progressive multiple sclerosis. However, we are not aware of any previous publications assessing total direct medical costs in patients with a long follow-up within the Brazilian healthcare system. Consequently, the objective is to analyze public spending on patients with MS to guide stakeholders in future investment and disinvestment decisions.

**Methods and findings:**

We retrospectively analyzed public Brazilian spending on patients with MS between 2000 and 2015 using the patient-centered registry of all patients in the public health system (SUS) obtained through deterministic-probabilistic record linkage of the Outpatient Information System, Hospital Information System and Mortality Information Systems in Brazil. Descriptive data analysis and a multiple linear regression model was performed to evaluate the associations between the mean annual cost per patient and the clinical and demographic variables. The suitability of the model was verified from a residue analysis and the level of significance adopted was 5%.

**Results:**

28,401 patients were identified and subsequently 23,082 patients were analyzed. The majority of the patients were female (73.3%), lived in the southeast region (58.9%), had a mean age of 36.8 (± 12.2) years and started treatment using one of the interferons beta (78.9%). The total direct medical cost spending in the sixteen years of the follow-up was US $ 2,308,393,465.60, and the mean annual expenditure per patient was US $ 13,544.40 (± 4,607.05). In the best fit model (p <0.001), approximately 40% of the variability of the mean annual cost per patient was explained by the region of residence; medication used (intention to treat); if the patient was a non-exclusive user of medicines, i.e., used SUS for other procedures other than high-cost medicines; year of treatment start; and presence of events (death; Relapse; change of treatment and/or comorbidity).

**Conclusions:**

In the public health system of Brazil, disease modifying therapies currently represent almost all of the total direct costs of multiple sclerosis treatment. Around the world, new and emerging health technologies to treat of MS impose a challenge to health budgets, highlighting the need for cost-effectiveness studies comparing these technologies to those already available. Our regression model may help in this process, and calls attention to the need to access the real world performance of new therapies available in SUS, with the potential for disinvestment and/ or price reductions if needed.

## Introduction

Multiple sclerosis (MS) is a chronic multifactorial disease, characterized by inflammation, demyelination and axonal loss, mainly in the white matter of the central nervous system. In most cases, the disease is manifested by neurologic acute symptoms that can be severe or seem so trivial that the patient may not seek medical care for months or years, usually followed by period of relief or absence of symptoms; hence, the relapse-remitting characteristic. Spasticity, optic neuritis, diplopia, paresis or paresthesia of limbs, dysfunctions of coordination and balance, myelitis, sphincter and cognitive-behavioral dysfunctions, alone or in combination, are the main symptoms [1]. It was estimated that in Brazil the average prevalence of the disease is 8.69 / 100,000 inhabitants [2]. Most cases occur in young adults between 20 and 50 years of age, being more frequent in whites and twice as common among women [3].

In general, the diagnosis of MS is based on the occurrence of two or more symptomatic episodes that should last more than 24 hours each and with different symptoms, separated by at least one month [1,4]. Radiological and laboratory exams, including magnetic resonance imaging, cerebrospinal fluid analysis and visual evoked potentials, which together contribute to the clinical evidence, are essential for the diagnosis and monitoring of MS and for treatment [4–7].

In Brazil, since the enactment of the Federal Constitution of 1988, the right to health is universal. Thus, the Brazilian public health system, Sistema Único de Saúde—SUS, provides pharmacological treatment; hospital and outpatient care for each Brazilian citizen [8], including multiple sclerosis patients diagnosed as relapsing-remitting or secondary progressive multiple sclerosis (RRMS and SPMS). SPMS includes patients with chronic advanced MS. In addition to the services offered by the SUS, patients can also choose to contract with private health plans (supplementary health) [9]. However, only just under a quarter of citizens in Brazil have private insurance 23.6% [10].

The pharmacological options in SUS comprise disease modifying therapies (DMT), usually high cost drugs including the beta interferons, glatiramer, natalizumab and fingolimod as well as low cost DMT such as azathioprine. Methylprednisolone is funded for the treatment of relapses, with medicines also funded for symptomatic relief [1].

In Brazil until 2010, DMT consisted of glatiramer acetate, interferon beta 1a and 1b and azathioprine. In the following years, natalizumab and fingolimod were added as treatment options from SUS [7,11,12]. However, DMT dispensing in SUS is conditioned on compliance to the Clinical Guideline of Ministry of Health, which is updated by the National Commission for Technology Incorporation in SUS, CONITEC [13]. This makes it easier to track patient level information for these patients. As a result, dispensing information regarding DMT and outpatient procedures, such as treatment for relapses and physiotherapy, are routinely recorded in the SUS Outpatient Information System. Hospital procedures, such as treatment for relapses and complications, are recorded in the SUS Hospital Information System. The integration of these databases, and the Mortality Information System, allows the construction of a robust patient-centered registry for long term longitudinal follow-up of these patients [14].

MS is a disease that stands out for its appreciable impact on quality of life, as well as the high cost associated with its treatment [15–17]. Kolasa carried out a systematic review of the literature that sought to measure the costs of MS treatment. Seventeen studies from 14 countries were included, of which 16 were retrospective studies based on questionnaires and one was based on patient records [18]. In most studies, the follow-up period did not exceed three months. The total direct cost per year per individual ranged from US $ 13,921 to US $ 54,600, presenting an average of US $ 41,133. Expenses with medicines accounted for 45.7% of direct costs, while 21.7% were spent on orthopedic appliances and adaptation measures, 15.2% in hospital care, 13.0% in outpatient care and 4.3% in exams [18].

In a cross-sectional study conducted in Brazil, the total mean annual cost was US $ 19,012. The cost of DMTs contributed the majority of direct expenditure, especially among those patients with lower levels of disability, accounting for approximately 90% of the total costs for mild and moderate MS patients [19]. Hawton and Green demonstrated that treatment expenditures are higher in patients who have outbreaks compared to patients who have not relapsed in the last six months, and expenditure increased substantially when relapses required hospital admission [20]. Most published cost effectiveness evaluations of the treatments for MS use long-term modeling; however, they typically use data from primary studies conducted for a short period only because of the paucity of studies performed over a long follow-up period][21].

In Brazil, despite ongoing constraints and the high cost of DMTs, the long-term spending on patients with MS within SUS is unknown. In 2015, The QuintilesIMS Institute published a report that highlights different aspects of the use of medicines in the United States (US), specially spanning overall spending and patient cost exposure. According to their report, the spend on DMTs for MS represented the eighth largest drug spending in US in 2014/2015 [22], endorsing the need to analyse these costs in Brazil for future policy analysis. Consequently, we believe it is mandatory to analyze actual SUS spending on patients with MS in Brazil, including long-term follow-up, to guide stakeholders in future investment and disinvestment decisions. This builds on the recently agreed process formalising disinvestment decisions in Brazil including medicines for multiple sclerosis [23,24]. We also hope our results will be useful to aid future cost-effectiveness analysis of treatments for multiple sclerosis in Brazil and wider.

## Methods

### Study design and population

We retrospectively analyzed the spending with MS between 2000 and 2015 using the patient-centered registry obtained through deterministic-probabilistic record linkage of the Outpatient Information System, Hospital Information System and Mortality Information Systems in SUS. The methodological procedures adopted for the relationship were the same ones described by Pereira *et al*. [25]. The monetary values were adjusted according to the purchasing power parity index (PPP) of the World Bank [26].

Patients were included if they were diagnosed with Multiple Sclerosis (G35) according to the tenth revision of the international classification of diseases (ICD-10) and started DMT between January 2000 and January 2015. During this period, the following DMT drugs were available through SUS: three presentations of interferon beta (βINF), two subcutaneous (SC) and one intramuscular (IM); glatiramer; natalizumab; fingolimod and azathioprine. We excluded from the study individuals whose data showed signs of errors during deterministic-probabilistic record linkage and patients who did not remain in the cohort for at least one year of observation. All patients were followed until the death or until December 2015 (right censoring). A one year follow-up period was seen as the minimum to start to accurately compute costs associated with MS.

### Costs analysis

The cost analysis took the perspective of the Brazilian Ministry of Health and was limited to the direct medical costs contained within the registry information systems, which includes the costs of medicines within the public system. Consequently, we did not include indirect costs in our analysis. Costs were classified in the following groups: DMT, treatment of relapses, diagnostic and monitoring exams, orthopedic appliances and rehabilitation, other outpatient services and other hospital services. We included laboratory examinations and visual evoked potentials as costs associated with monitoring disease progression. The total cost and the relative frequency were calculated for each category. To describe the evolution of expenditures according to the year of follow-up (1st year of treatment, 2nd year of treatment, and so on), the average annual cost per patient was calculated for each of the groups.

The total annual cost per patient was calculated by adding the amount spent on drugs, outpatient and hospital services for each follow-up year. The mean annual cost per patient, determined by the central tendency measure of the individual annualized costs, was calculated for each variable of interest. These were gender, age, region of residence, and the medicines used for multiple sclerosis at the start of treatment and calendar period in which the patient entered the registry. We also summarized data on the main comorbidities, and occurrence of relapses, change of drug during the follow-up period and cause of death. Relapse was defined by the occurrence of methylprednisolone dispensing and/or pulse steroid therapy and/or hospital relapse treatment. For the identification of comorbidities, we used the algorithm developed by Hude Quan *et al*. [27].

### Statistical analysis

Descriptive data analysis was performed calculating the frequency distribution for categorical variables and measures of central tendency and variability for continuous variables. A multiple linear regression model was performed to evaluate the associations between the mean annual cost per patient and the clinical and demographic variables. We log-transformed the mean annual expenditure and employed the least squares technique to find the best fit for the dataset. The suitability of the model was verified from a residue analysis and the level of significance adopted was 5%. We performed an additional analysis considering that there are two types of patients in the SUS: (i) the user of the public health system who accesses various services offered which include outpatient and hospital procedures, and (ii) the patient who uses SUS only to get high-cost drugs and perform the remainder of the treatment through their private health plans. The second multiple regression model was constructed from the data of the first group (i) and aimed to perform a sensitivity analysis. We used the variance inflation factor (VIF) to rule out the presence of multicollinearity in the model. All analyzes was performed using R Program 3.4.0 and RStudio 1.0.143.

This research was approved by the Research and Ethics Committee of the Universidade Federal de Minas Gerais (n° 1.072.253).

## Results

**Fig 1:**
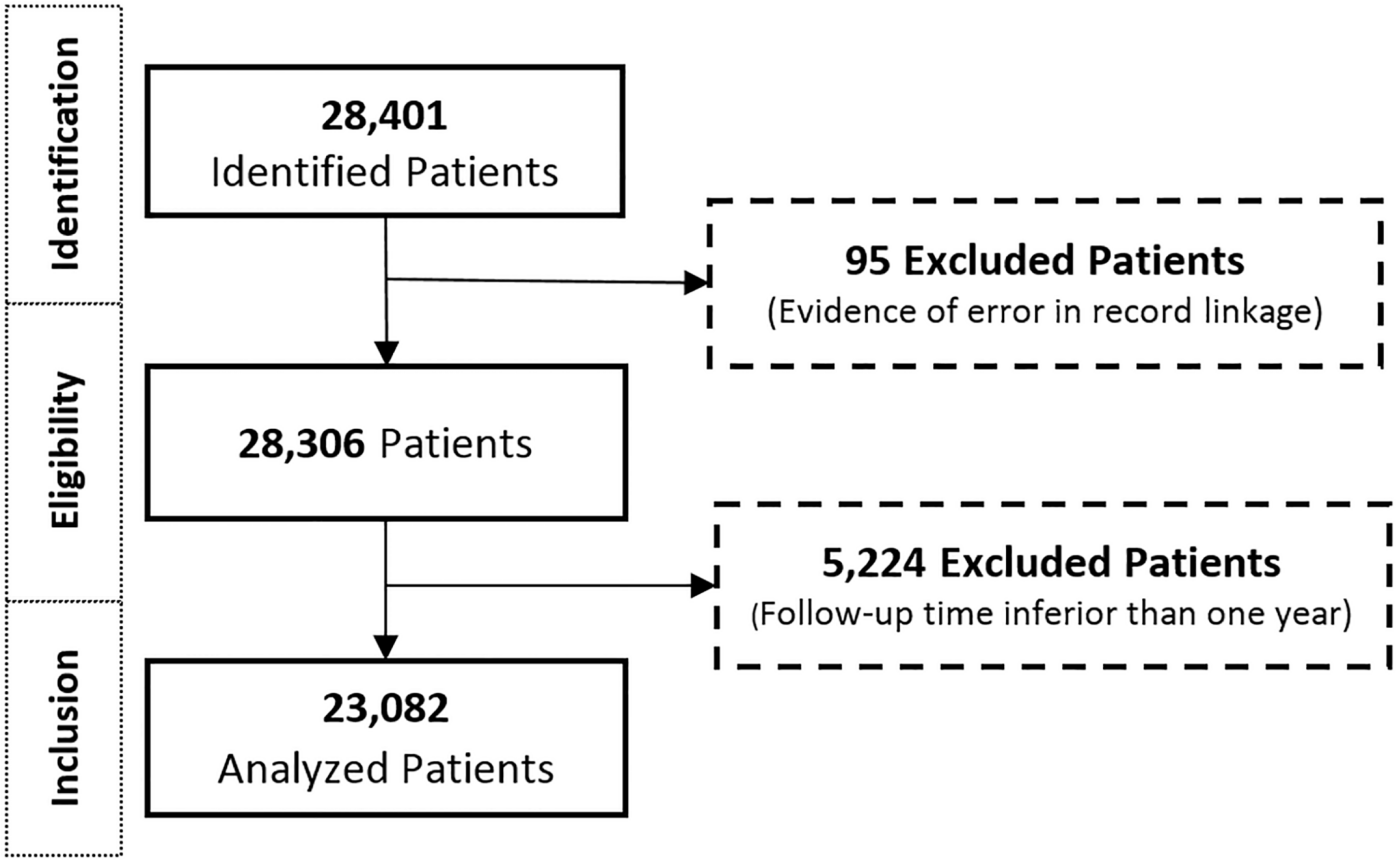
Patient disposition flow diagram. Patient disposition flow diagram showing patients included and excluded from the study cohort.

We identified 28,401 patients within the dispensing registry who had at least one of the DMTs for the treatment in the period 2000 to 2015. Of these, we excluded 95 (0.33%) patients whose data indicated an error during the deterministic-probabilistic record linkage and 5,224 patients who did not remain in the cohort for at least one year. At the end, 23,082 patients were analyzed (Fig 1).

The majority of the patients included in the study were female (73.3%), lived in the southeast region (58.9%), had a mean age of 36.8 (± 12.2) years and started treatment using one of the beta interferons (78.9%). The first prescribed DMT was discontinued and changed in 9,835 patients (42.6%). When we analyzed the comorbidities and deaths, we verified that 5,088 (22.0%) patients had a history of one or more comorbidities during the study period, in which other neurological diseases, paralysis and rheumatoid arthritis were the most frequent. We found that 1,135 (4.9%) deaths were recorded and the main declared cause was the disease itself (38.59). The total spending on medicines, outpatient and hospital services in the sixteen years of the follow-up was US $ 2,308,393,465.60, and the mean annual expenditure per patient was US $ 13,544.40 (± 4,607.05). The annual cost per patient with high-cost drugs, hospital and outpatient services, distributed according to the clinical and demographic variables of the population are reported in Table 1.

**Table 1:** Mean annual cost per patient according to clinical and demographic variables, DMT drug at study entry and sequence of events for the 23,082 MS patients. Brazil: 2000–2015.

Variables		N (%)	Mean annual cost per patient (US $, SD)
Cohort		23082 (100.00)	13.544.40 (± 4.607.05)
Gender	Female	16919 (73.30)	13,537.81 (± 4,590.97)
	Male	6163 (26.70)	13,561.23 (± 4,664.22)
Age	0–17	833 (3.61)	12,295.33 (± 4,001.04)
	18–25	3359 (14.55)	13,270.51 (± 4,449.04)
	26–35	6988 (30.27)	13,346.65 (± 4,573.04)
	36–45	6216 (26.93)	14,273.69 (± 4,836.83)
	46–55	4163 (18.04)	13,478.47 (± 4,806.85)
	56–65	1280 (5.55)	12,322.51 (± 4,319.04)
	> 65	243 (1.05)	12,103.21 (± 4,366.05)
Geographic region	Southeast	13605 (58.94)	13,834.53 (± 4,985.88)
	South	4458 (19.31)	12,644.44 (± 4,376.67)
	Northeast	2644 (11.45)	12,727.25 (± 4,636.75)
	Midweast	2025 (8.77)	13,964.30 (± 4,688.50)
	North	350 (1.52)	11,233.12 (± 4,363.80)
DMT (at start of treatment)	Subcutaneos interferon beta 1a (Rebif^™^)	7881 (34.14)	16,913.10 (± 6,799.14)
	Intramuscular interferon beta 1a (Avonex^™^)	5450 (23.61)	11,522.19 (± 4,489.08)
	Subcuaneous interferon beta 1b (Betaferon^™^ or Extavia^™^)	4886 (21.17)	12,661.66 (± 4,251.33)
	Glatiramer (Copaxone^™^)	3953 (17.13)	9,659.48 (± 3,772.44)
	Azathioprine	672 (2.91)	4,822.24 (± 2,652.35)
	Natalizumab (Tysabri^™^)	144 (0.62)	8,668.26 (± 4,651.24)
	Other DMT Combinations	96 (0.42)	10,909.20 (± 5,090.78)
Period of study entry	2000 a 2003	4611 (19.98)	18,239.78 (± 7,959.18)
	2004 a 2007	5039 (21.83)	14,943.18 (± 7,411.10)
	2008 a 2011	8070 (34.96)	11,528.00 (± 5,574.57)
	2012 a 2015	5362 (23.23)	8,324.58 (± 3,268.17)
Events	Censorship	11888 (51.50)	12,810.40 (± 4,218.30)
	Treatment failure (global)	11194 (48.50)	14,098.78 (± 5,099.70)
	Treatment failure (relapses)	2171 (9.41)	12,664.85 (± 5,684.52)
	Treatment failure (switched the medication)	9835 (42.60)	14,215.30 (± 5,133.79)
	Treatment failure (death)	1135 (4.92)	13,800.46 (± 5,837.92)
Cause of Death (ICD-10)	Multiple Sclerosis (G35)	438 (38.59)	10,475.94 (± 6,316.14)
	Acute myocardial infarction (I219)	37 (3.26)	15,299.95 (± 4,984.23)
	Other disorders of the urinary tract (N390)	26 (2.29)	14,993.01 (± 6,573.97)
	Other septicemia (A419)	25 (2.20)	12,344.86 (± 5,435.85)
	Pneumonia unspecified (J189)	24 (2.11)	19,197.85 (± 6,675.30)
	Other Causes	585 (51.54)	12,923.32 (± 4,309.42)
Comorbidity*	Paralysis	1312 (18.77)	12,166.62 (± 5,564.66)
	Rheumatoid arthritis/collagen vascular diseases	912 (13.05)	12,412.63 (± 6,516.65)
	Liver disease	543 (7.77)	16,164.83 (± 5,823.15)
	Renal failure	461 (6.60)	16,897.48 (± 7,955.72)
	Psychoses	321 (4.59)	14,142.52 (± 5,216.06)
	Depression	122 (1.75)	12,029.42 (± 4,999.55)
	Other neurological disorders	1959 (28.04)	11,680.16 (± 5,532.18)
	Others Comorbidities	1357 (19.42)	13,379.96 (± 6,949.39)

* The ICD-10 coding algorithms for these comorbidities are shown in Hude Quan *et al* study [27].

**Fig 2:**
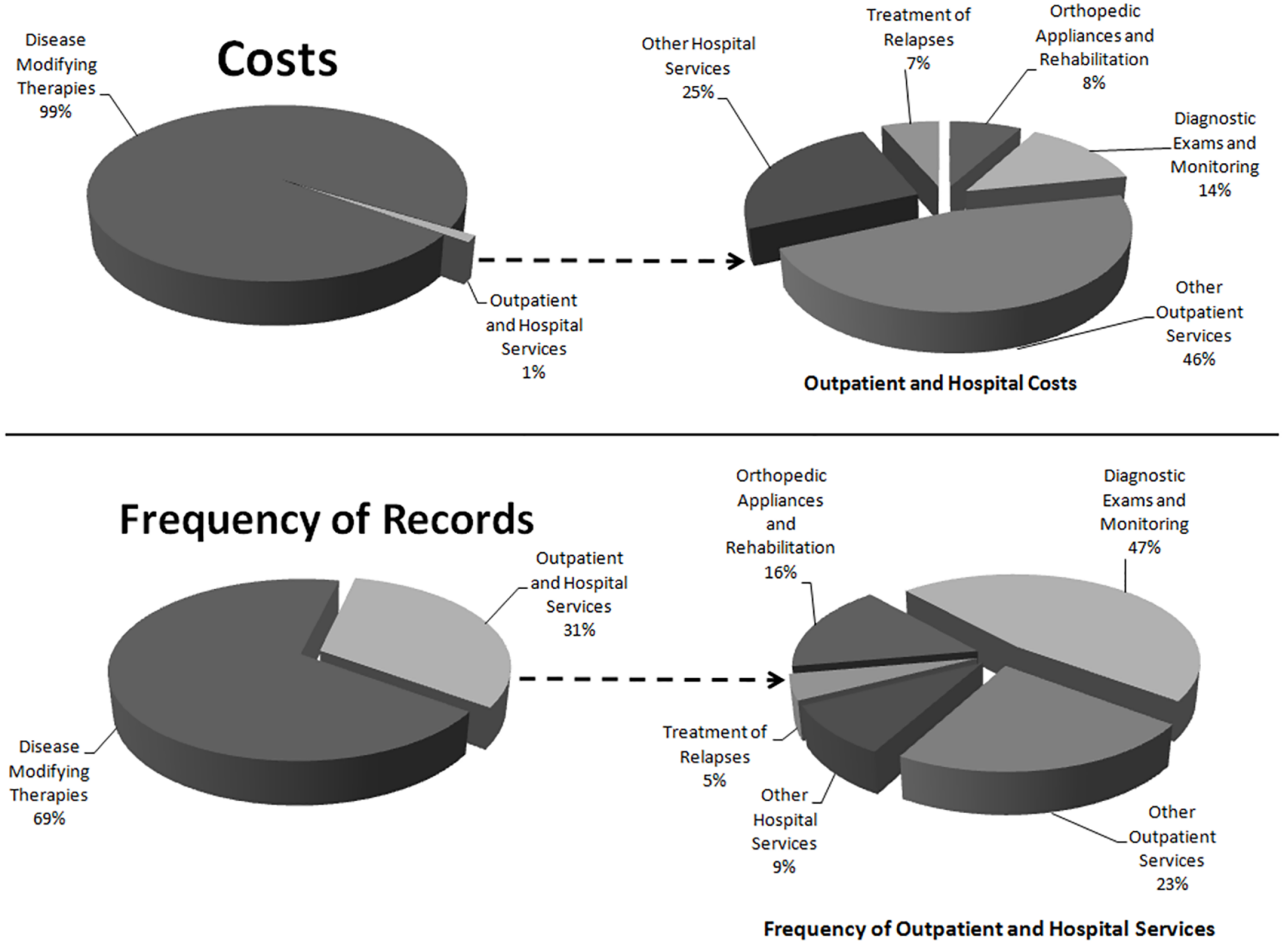
Total average cost and relative frequency of services in the MS cohort.

The cost of DMTs accounted for 99% of the total cost, and most patients (56.1%) used SUS only to have access to high-cost drugs. DMT dispensing accounted for 69% of all procedures captured in the registry. Diagnostic examinations and monitoring were the services most frequently used (47%), although spending on these procedures represented only 14% of the total cost. Magnetic resonance imaging was the most common examination, representing the higher relative frequency in this category (45.5%) (Fig 2). Fig 2 combines the average costs for each service building on Table 1 (which includes information on SDs).

When we analyzed the other categories, we verified that physiotherapy (52.9%) was the service with the highest relative frequency in "orthopedic appliances and rehabilitation", as was the case with the use of antiepileptic drugs (11.5%), clinical consultations (9.2%) and use of peripheral muscular relaxants (8.5%) in the category "other outpatient services". In "other hospital services", clinical hospital care (50.9%) and surgeries (30.0%) were the most frequent services.

**Fig 3:**
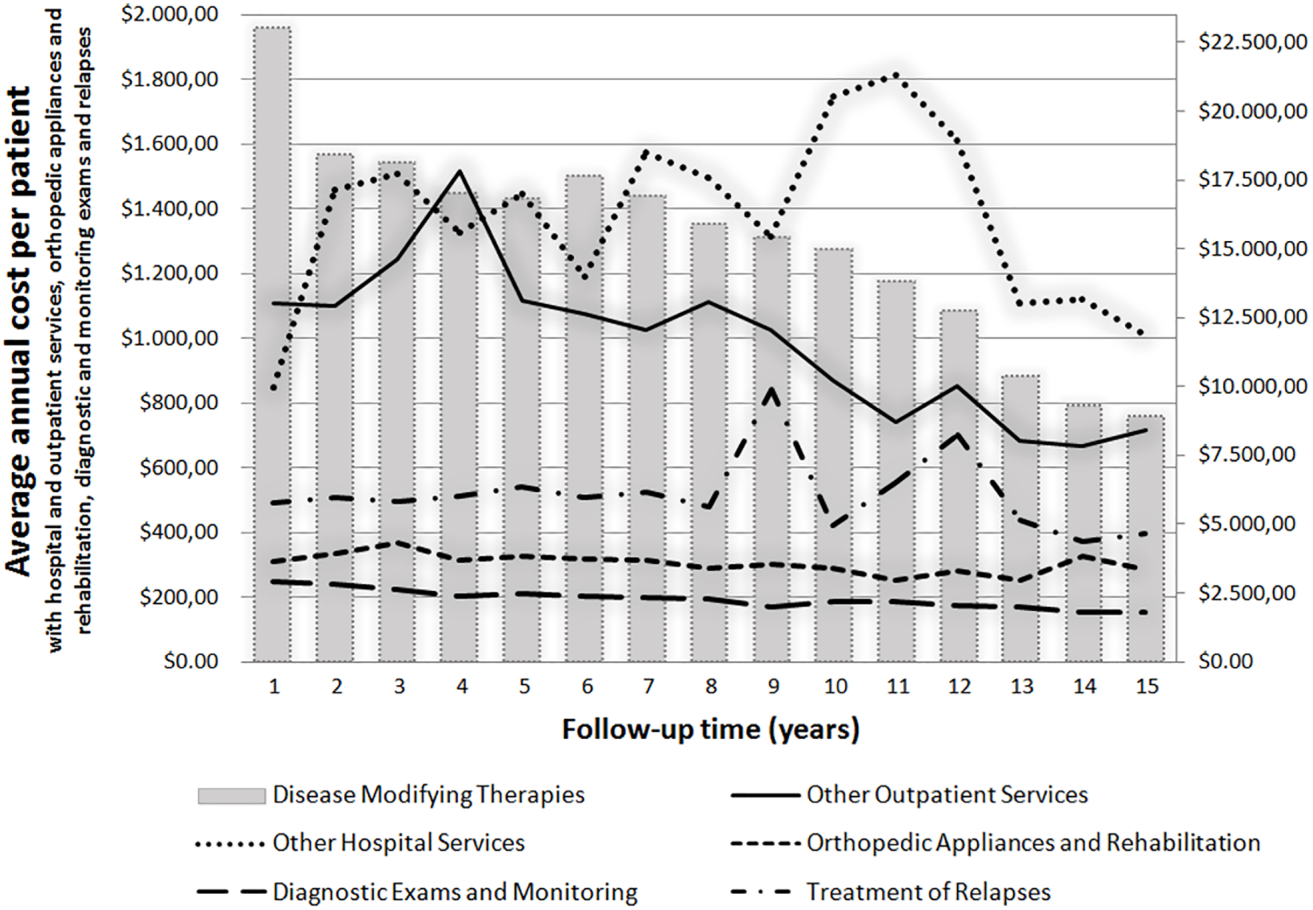
Average annual cost per patient and follow-up time, adjusted by PPP index.

During the follow-up years, there was a decrease in the mean annual cost per patient with DMTs. In contrast, spending on hospital services and the treatment of relapses showed a growth trend over the years (Fig 3).

In the best fit model (p <0.001), approximately 61% (Multiple R-squared: 0.6109, Adjusted R-squared: 0.6106) of the variability of the mean annual cost per patient was explained by the region of residence; medication used (intention to treat); if the patient was a non-exclusive user of medicines, i.e., used SUS for other procedures beyond high-cost drugs dispensed; year of the start of treatment; and presence of events (death, relapse, change of treatment and/or comorbidity) (Table 2).

**Table 2:** Multiple linear regression model with multiple sclerosis patients included in the cohort, 2000 to 2015, Brazil*.

Predictive variables	B	Standard Error	t value	p value
Intercept	8.26E+28	2.036443	32.696	<0.001
South	1.08	0.024993	2.980	0.003
Northeast	1.06	0.025564	2.155	0.031
Southeast	1.16	0.024362	6.146	<0.001
Midwest	1.18	0.026016	6.486	<0.001
Intramscular interfeon beta 1a (Rebif^™^)	1.18	0.008109	20.376	<0.001
Subcuaneous interferona beta 1b (Betaferon^™^ or Extavia^™^)	0.92	0.008959	-9.174	<0.001
Glatiramer (Copaxone^™^)	0.88	0.009323	-13.622	<0.001
Natalizumab (Tysabri^™^)	1.11	0.037658	2.678	0.007
Azathioprine	0.06	0.018576	-150.543	<0.001
Other DMT Combinations	0.95	0.046094	-1.111	0.267
Non-exclusive user of medicines	0.89	0.006598	-17.566	<0.001
Year of treatment start	0.97	0.001012	-28.133	<0.001
Treatment time	1.02	0.001159	19.903	<0.001
Registry of comorbidity	0.93	0.007957	-9.187	<0.001
Treatment failure by relapse	1.01	0.017041	0.586	0.558
Treatment failure by switched medication	0.93	0.006856	-10.409	<0.001
Treatment failure by death	1.08	0.020965	3.464	0.001
Treatment failure by relapse and switched medication	0.95	0.014243	-3.889	<0.001
Treatment failure by relapse and death	1.29	0.048735	5.299	<0.001
Treatment failure by switched medication and death	0.95	0.023377	-2.021	0.043
Treatment failure by relapse, switched medication and death	1.04	0.039441	1.003	0.316

* 309 (1,3%) outliers were excluded from the model after residue analysis

They were excluded because they showed signs of errors during the parameter data, altering in a significant way the average of the data. However, since outliers do not represent a population, we preferred to exclude them from analysis.

Using SC βINF-1a and natalizumab at the start of treatment increased by 18% and 11%, respectively, the mean annual cost per patient compared to patients using IM βINF-1a. Having used SC βINF-1b, glatiramer or azathioprine, reduced the cost by 8%, 12% and 99%, respectively, compared to the use of IM βINF-1a, with the other variables constant. The increase in the mean annual cost per patient was inversely proportional to the year of start of treatment, but directly proportional to the time of treatment.

When analyzing treatment failures, in general the presence of any event, as well relapse, relapse followed by death, death and when the patient has a record of relapse, switched medication and death, increased the mean annual expenditure when compared to patients who did not experience any of these events. In turn, patients who had a switched medication record showed a reduction of expenditure by 7%. The same occurred for patients who switched their medication and died, and those who had a relapse and switched medication, who had a reduction of spending by 5%.

Patients living in the south, northeast, southeast and midwest regions of Brazil had an average annual cost per patient higher than the patients in the northern region of Brazil. Patients in the midwest region (p <0.001) contributed most to this increase in comparison to others, since this region showed an increase in spending of 18% compared to the northern region of Brazil. Patients who used hospital and outpatient services, in addition to high-cost medicines, had lower annual costs per patient than patients who accessed SUS only to obtain drug treatment (p <0.001).

In the sensitivity analysis, focusing on patients that used SUS for more than just medication access, the best fit model (p <0.001) showed that approximately 60% (Multiple R-squared: 0.6056, Adjusted R-squared: 0.6053) of the variability of the mean annual cost per patient was explained by the same predictive variables of the first model (Table 3).

**Table 3:** Sensitivity analysis of multiple linear regression model with non-exclusive user of medicines. 2000 to 2015.

Predictive variables	B	Standard Error	t value	p value
Intercept	3,18E+29	2.048.707	33.158	< 0.001
South	1,09	0.025158	3.255	0.001
Northeast	1,07	0.025732	2.463	0.014
Southeast	1,18	0.024516	6.611	< 0.001
Midwest	1,19	0.026189	6.663	< 0.001
Intramscular interfeon beta 1a (Rebif^™^)	1,17	0.008160	19.714	< 0.001
Subcuaneous interferona beta 1b (Betaferon^™^ or Extavia^™^)	0,91	0.009006	-10.076	< 0.001
Glatiramer (Copaxone^™^)	0,88	0.009384	-13.880	< 0.001
Natalizumab (Tysabri^™^)	1,10	0.037908	2.418	0.016
Azathioprine	0,06	0.018689	-150.254	< 0.001
Other DMT Combinations	0,95	0.046405	-1.109	0.268
Year of treatment start	0,97	0.001018	-28.644	< 0.001
Treatment time	1,02	0.001166	19.145	< 0.001
Registry of comorbidity	0,89	0.007546	-15.903	< 0.001
Treatment failure by relapse	0,97	0.016993	-1.836	0.066
Treatment failure by switched medication	0,93	0.006902	-10.424	< 0.001
Treatment failure by death	1,06	0.021090	2.749	0.006
Treatment failure by relapse and switched medication	0,91	0.014159	-6.697	< 0.001
Treatment failure by relapse and death	1,24	0.049005	4.420	< 0.001
Treatment failure by switched medication and death	0,94	0.023526	-2.499	0.012
Treatment failure by relapse. switched medication and death	1,00	0.039628	-0.106	0.915

The results in the analysis with the restricted group were very similar to the original model for almost all variables (DMT, region of residence, year of treatment start, treatment time and registry of comorbidities), changing only the value of the beta coefficient. However, when analyzing the events that may indicate some failure of the treatment, we verified that unlike the first mode the expenditure with patients who presented a relapse is lower by 3% compared to those who did not present any event. However, it is important to note that this result was not significant (p = 0.066).

## Discussion

The costs of treating patients diagnosed with MS were influenced by clinical and demographic factors. When we analyze the mean annual cost per patient, the costs varied according to the region, health policy and type of methodology employed. Several studies have presented similar costs to those recorded in our cohort (US $ 13,544), such as the results found by Prescott et al (US $ 12,879), Silva et al (US $ 19,012.32) and Palmer et al when just direct costs are considered [20,28,29]. However, Curkendall *et al* suggested higher annual costs between $28,280 and $29,102 depending on the stage of the MS [30]. Patti et al also suggested higher annual costs at €18,030/ patient.[31]. Kolbet et al also found that costs are dependent on the availability, use and price of services and on disease severity. All of these varied between countries, leading to very different mean annual costs per patient. In their study, overall mean costs (in € PPP) for patients with mild, moderate and severe disease were €22,800 (range of country means, 12,600–27,300), 37,100 (22,500–54,700) and 57,500 (27,500–77,600), respectively [32]. Consequently, it is important to put any costs into context.

The majority of the population studied was female (73.3%). This is perhaps not surprising as multiple sclerosis is twice as common among women than men [3,31]. The reason for this difference is not fully understood, although it is likely to be associated with socioenvironmental factors, with underlying genetic differences [1].

Almost eighty percent of the patients started treatment with one of the beta interferons, which is in accordance with the Brazilian clinical protocol funded within SUS. Until 2017, these drugs and glatiramer were the only ones provided by the Brazilian public health as first line of treatment of multiple sclerosis. [7,12,14]. This utilisation pattern was also observed by Moccia *et al*. that showed SC βINF-1a was the most prescribed drug for patients with MS in the 10-year Italian cohort (32.1%), followed by IM βINF-1a (19.7%) and SC βINF-1b (16.6% %). This same study also observed that 53.4% of the patients discontinued the treatment and switched medication, which was similar to our findings (42.6%) [33].

The most common cause of death in MS patients was "MS" (38.6%). Similar results were found by Jick *et al* [34] in which 41% of patients who died had their own disease reported as death. The second most common cause of death reported in our study patients was acute myocardial infarction (n = 37). When we evaluated the other comorbidities not listed in Table 1 but related to cardiovascular problems, we found that 83 patients reported congestive heart failure, 25 have cardiac arrhythmias and 25 reported valvular disease.

Studies have also shown that DMTs represent the greater proportion of direct costs within the treatment of MS, followed by medical consultations and physiotherapy; the latter the most frequent procedure [35]. DMTs accounted for 99% of healthcare costs in our study, consistent with the finding by Kolasa et al, which reported that the most important contributions to direct medical costs were the costs of the medicines [18]. However, appreciably higher than the 40–46% in the analysis by Curkendall et al depending on the stage of MS and 61–73% in the study by Patti et al depending on MS type [30,31]. According to the study by Ernstsson O et al, drugs were the main cost drivers for MS-patients with low disease severity (29% to 82% of all costs), while the main cost components for groups with more advanced MS symptoms were production losses due to MS and informal care (17% to 67%) [36]. As mentioned, we did not include indirect costs in our study as we took the perspective of the Brazilian healthcare system. The high proportion of costs for drug treatment in our study can be explained by the high cost of these medicines relative to other healthcare costs and the fact that a number of patients, especially those with private insurance, do not use SUS for their follow-up and any other medical needs—just the cost of the medicines. In Brazil, all citizens are covered by SUS; however, they can purchase individual or work class health plans for outpatient and hospital care [9]. Usually high-cost drugs are not included in this modality of health assistance, for instance, no DMT is currently provided by private health plans; consequently, the high proportion of patients accessing SUS only to have access to these medicines [10]. As a result, there is typically no record of relapse treatment or other medical assistance in the SUS Information Systems for these patients influencing our findings.

When analyzing the results of multiple linear regression, the greatest expense associated that the use of SC INFβ-1a or natalizumab at the start of treatment has a direct relationship with the high cost of these medicines to the Brazilian Ministry of Health. SC INFβ-1a has the highest unit value (government acquisition price) in our database, and consequently the exchange for any other drug in the cohort leads to a decrease in the relative cost. Regarding the region of residence, higher costs were associated with more developed states where there is a greater number of qualified professionals and better organizational structure, resulting in higher availability of doctors, hospitals, outpatient clinics and financial resources. These regions are the richest and present the best Human Development Index (HDI) in Brazil[37]. In the first regression model with all patients in the cohort, the presence of any event increases the mean annual cost per patient. The same occurred in the sensitivity analysis with patients accessing SUS for more than DMT, indicating that, in fact, the more complicated the patient, the higher the overall cost of treatment.

Recently, there have been debates about medicine incorporation for patients with MS within SUS [38–40]. As mentioned, first-generation DMTs IFNβ and glatiramer acetate were incorporated in SUS in 2001 as first line of treatment; natalizumab was incorporated in 2010 as second line and fingolimod was incorporated in 2014 as third line treatment [7,11,12]. However, there has been a decision to disinvest in IM INF-1a SUS due to its proven inferiority compared to other pharmaceutical interferon presentations [24, 36]. In 2017, teriflunomide was incorporated as a first line option for treatment of RRMS after the recommendation of CONITEC [39]. Following this, fingolimode was also approved for second line of treatment [39]. Hopefully, spending analyses such as ours will contribute to a more accurate prediction of future expenditures of patients with MS in Brazil and other countries and helps the Brazilian healthcare system adapt to these recent changes. We will be monitoring this in the future.

One of the limitations of this study is related to the fact that the information systems used to compose the registry under study are for administrative purposes. This means that some clinical information is missing, such as time of diagnosis, Expanded Disability Status Scale (EDSS) and reasons for DTM switching. This is the same though for most administrative databases. This will be the subject of future research projects in Brazil. It is also important to consider the possibility of incorrect data being incorporated into the system that may culminate in the sub or overestimation of costs. In addition, as mentioned, some aspects of care are not included if patients purchased individual private insurance plans or had access to private insurance via their workplace. In an attempt to minimize possible inconsistencies, after the data collection, a sample inspection was performed with subsequent cleaning and standardization, important stages to guarantee the quality of the collected data. Consequently, we believe our findings are robust regarding direct medical costs. We are aware that we did not include indirect costs with authors suggesting that these costs are similar to direct medical costs. However, our focus was SUS and only direct medical costs.

## Conclusions

In the public health system of Brazil, DMTs comprise almost all of the total direct costs of multiple sclerosis treatment. Around the world, new and emerging health technologies are becoming available to treat of MS. These are typically more expensive than existing treatment, imposing a challenge to health budgets. As a result, highlighting the need for cost-effectiveness studies compared to available technologies. We believe our regression model of costs may help in this process, and calls attention the need to access the real world performance of DTMs within public healthcare systems to guide future decisions. These can be for either future investment or disinvestment decisions as well as for future price negotiations within public systems.

## Public sharing of data

The SUS data base is not open for public review with strict regulations and requirements for access. Researchers can contact the authors for more information if wished regarding the analysis.

## Abbreviations

CONITEC: National Commission for Technology Incorporation in SUS
DMT: disease modifying therapies
EDSS: Expanded Disability Status Scale
ICD: international classification of diseases
IM: intramuscular
MS: multiple sclerosis
PPP: purchasing power parity index
RRMS: relapsing-remitting multiple sclerosis
SC: subcutaneous
SPMS: secondary progressive multiple sclerosis
SUS: Sistema Único de Saúde
βINF: interferon beta
